# Transcriptional Activation and Cell Cycle Block Are the Keys for 5-Fluorouracil Induced Up-Regulation of Human Thymidylate Synthase Expression

**DOI:** 10.1371/journal.pone.0047318

**Published:** 2012-10-09

**Authors:** Alessio Ligabue, Gaetano Marverti, Ursula Liebl, Hannu Myllykallio

**Affiliations:** 1 INSERM U696, Palaiseau, France; 2 Laboratoire d'Optique et Biosciences, CNRS, Ecole Polytechnique, Palaiseau, France; 3 Dipartimento di Scienze Biomediche, Sezione di Chimica Biologica, University of Modena and Reggio Emilia, Modena, Italy; University of Texas MD Anderson Cancer Center, United States of America

## Abstract

**Background:**

5-fluorouracil, a commonly used chemotherapeutic agent, up-regulates expression of human thymidylate synthase (hTS). Several different regulatory mechanisms have been proposed to mediate this up-regulation in distinct cell lines, but their specific contributions in a single cell line have not been investigated to date. We have established the relative contributions of these previously proposed regulatory mechanisms in the ovarian cancer cell line 2008 and the corresponding cisplatin-resistant and 5-FU cross-resistant-subline C13*.

**Methodology/Principal Findings:**

Using RNA polymerase II inhibitor DRB treated cell cultures, we showed that 70–80% of up-regulation of hTS results from transcriptional activation of TYMS mRNA. Moreover, we report that 5-FU compromises the cell cycle by blocking the 2008 and C13* cell lines in the S phase. As previous work has established that TYMS mRNA is synthesized in the S and G_1_ phase and hTS is localized in the nuclei during S and G_2_-M phase, the observed cell cycle changes are also expected to affect the intracellular regulation of hTS. Our data also suggest that the inhibition of the catalytic activity of hTS and the up-regulation of the hTS protein level are not causally linked, as the inactivated ternary complex, formed by hTS, deoxyuridine monophosphate and methylenetetrahydrofolate, was detected already 3 hours after 5-FU exposure, whereas substantial increase in global TS levels was detected only after 24 hours.

**Conclusions/Significance:**

Altogether, our data indicate that constitutive TYMS mRNA transcription, cell cycle-induced hTS regulation and hTS enzyme stability are the three key mechanisms responsible for 5-fluorouracil induced up-regulation of human thymidylate synthase expression in the two ovarian cancer cell lines studied. As these three independent regulatory phenomena occur in a precise order, our work provides a feasible rationale for earlier observed synergistic combinations of 5-FU with other drugs and may suggest novel therapeutic strategies.

## Introduction

Human thymidylate synthase of the ThyA family [hTS (EC 2.1.1.45), encoded by the gene TYMS] is a folate-dependent enzyme that converts 2′-deoxyuridine-5′-monophosphate (dUMP) and N^5^-N^10^-methylenetetrahydrofolate (mTHF) to dihydrofolate and 2′-deoxythymidine-5′-monophosphate (dTMP). Recent papers demonstrated that hTS is localized not only in the cytoplasm, but also in the nuclei and in the mitochondria. Nuclear hTS is associated with proliferating cell nuclear antigen (PCNA) and other components of the DNA replication machinery, suggesting that *de novo* thymidylate biosynthesis occurs at replication forks [1]. On the other hand, mitochondrial hTS prevents uracil accumulation in mitochondrial DNA and is essential for mtDNA integrity [2]. Human cells do not possess the flavin-dependent thymidylate synthase ThyX that is found in many free living microbes [3]. Consequently, hTS provides the only *de novo* pathway for thymidylate synthesis in human cells and represents an essential target enzyme for cancer chemotherapy [4]. Several inhibitors that prevent the catalytic activity of human thymidylate synthase through binding to dUMP and/or mTHF binding pockets have been identified. For instance, the uracil- analog 5-fluorouracil (5-FU), after metabolic conversion to 5-FdUMP, is a well characterized active-site inhibitor of hTS that has been widely used in chemotherapy since 1957 [5]. FdUMP forms a covalent ternary complex with hTS and mTHF, resulting in the irreversible inhibition of the catalytic activity of hTS. Inhibition of hTS provokes an increase of the intracellular dUMP concentration [6], [7] and causes depletion of deoxythymidine triphosphate (dTTP) [8]. The imbalance of intracellular deoxynucleotide pools disrupts DNA replication and triggers cell death [9], [10]. In addition to direct inhibition of hTS, the 5-FU metabolites 5-fluorouridine-5′-triphosphate (F-UTP) and 5-fluoro-2′-deoxyuridine-5′-triphosphate (FdUTP) cause cell death through incorporation into RNA and DNA, respectively (for a review see [11]). To prevent formation of resistant cell populations and to improve the response rate of treatment, 5-FU is usually given in combination with other drugs in clinical settings. For instance, a combination of 5-FU with irinotecan and oxaliplatin has increased the response rate to treatment for advanced colorectal cancer from 10%–15% to 40%–50% [12], [13], and histone deacetylase (HDAC) inhibitors have shown synergistic effects in combination with 5-FU [14], [15].

Although the reliability of thymidylate synthase expression as a clinical predictor of the response to 5-FU remains controversial [16], [17], it should be noted that the nuclear to cytosolic expression ratio of hTS predicts the outcome of 5-FU treatment better than the overall expression level [18]. It is well established that 5-FU administration increases the steady-state expression level of hTS in tissues and cell lines (for a review see [19]). Different regulatory mechanisms contributing to this phenomenon have been described in distinct human cell lines. For instance, in human gastrointestinal cell lines (Hutu 80, HT-29 and WIDR), as well as in human ovarian carcinoma cell lines (2008 and C13*), the ternary complex 5-FdUMP-MTF-hTS has increased stability as compared with the non-complexed enzyme, thus increasing up to 6-fold the steady-state expression level of hTS [20], [21]. The increase in protein stability is controlled by the amino-terminus of hTS that contains an intrinsically disordered region essential for ubiquitin-independent degradation by the proteasome and which may be partially buried in the ternary complex [22]. Kitchens [23] proposed the observed enzyme stabilization to be the primary mechanism that contributes to increased expression levels of hTS in human colon cancer cell lines (HTC15 and HTC15/200). In human hepatocellular carcinoma cell lines (Hep-AEG-1-14 and QGY-7703), over-expression of the transcription factor LSF, involved in G_1_/S phase transition of the cell cycle [24], increases the expression levels of hTS and 5-FU catabolic enzymes, thus partially conferring resistance to 5-FU [25]. It has also been proposed that in human colon cancer cells (H630 and H630R10), hTS binds to its own mRNA, resulting in translational repression [26], [27], [28]. In the presence of hTS ligands, including 5-FU and other active site inhibitors, the negative regulatory function of hTS as RNA binding protein is lost, resulting in increased expression. Finally, another mechanism of post-transcriptional regulation has been proposed in colorectal cancer cell lines (RKO, LoVo, DLD1 and SW620), where miRNA-192 and miRNA-195 modulate the expression levels of the TS protein without decreasing TYMS mRNA levels [29]. As far as we are aware, the possibility that incorporation of 5-FU into RNA may inhibit synthesis, stability and/or splicing of mature TYMS mRNA has not been addressed to date.

In this study we have investigated the relevance of these proposed mechanisms for hTS regulation and assayed their kinetics during 5-FU treatment in the ovarian cancer cell line 2008 and the corresponding cisplatin resistant- and 5-FU cross-resistant subline C13* that shows higher steady-state expression level of the enzymes of the folate cycle [20]. Despite the constitutively higher levels of TYMS in the C13* cell line, we found no obvious effect on splicing or maturation of TYMS pre-mRNA. Our findings support that in these cell lines a combination of increased protein stability and TYMS mRNA transcription (i.e. constitutive and 5-FU induced) is sufficient to increase hTS expression levels during 5-FU treatment. Our data addresses for the first time the relative contributions of these mechanisms involved in TS regulation in two distinct cell lines and may help to predict the observed synergistic effects between 5-FU and other drugs acting either on cell cycle regulation or on the stability of hTS.

## Materials and Methods

### Cell lines

The 2008 cell line was established from a patient with serious cystadenocarcinoma of the ovary and the cDDP-resistant C13* subline, about 15-fold resistant to cDDP and 2.5-fold cross-resistant to 5-FU, was derived from the parent 2008 cell line by 13 monthly selections where the cells were exposed chronically to cDDP starting at 0.25 µM (first month) and incrementally increased to 5.25 µM (last month) [30]. These human ovarian cell lines were grown as monolayers in RPMI 1640 medium containing 10% heat-inactivated fetal bovine serum and 50 µg/ml gentamycin sulfate. Cultures were equilibrated with humidified 5% CO_2_ in air at 37°C. Protein content in the various assays was calculated by the method of Bradford [31].

### Volume size determination and intracellular concentration of hTS

Cells were harvested and placed on a Burker cell counter and 100 randomly selected cells were examined using an Axioscope 40 epifluorescence microscope (Zeiss, Germany). The diameters were quantified at a 400-fold magnification by image analysis software (Axiovision 3.1 from Zeiss). The volume of a single cell was determined using the volume formula of the sphere. The intracellular concentration of mRNAs was established using retro transcription real-time PCR (RT-PCR) accounting for the aqueous phase volume recovered during tri-reagent extraction (80%). The real-time PCR amplification efficiency (100%) was determined according to the “Guide to Performing Relative Quantification of Gene Expression Using Real–Time quantitative PCR” (Applied Biosystems) and retro transcription efficiency (34%) was evaluated by comparing our condition with the condition already reported by Stahlberg et al. [32].

### Cell cycle analysis

Quantitative measures of the cell cycle phase distribution were performed by flow cytometry [33]. Cells were incubated with 10 µM BrdU for 1 h at 37°C and labeled with monoclonal anti-5-bromodeoxyuridine (Clone MoBu-1,Sigma) in conjunction with a goat anti mouse IgG-FICT (Fab*specific, Sigma). Subsequently, cells were suspended in 0.5 ml of hypotonic fluorochrome solution (50 µg/ml PI, 0.1% sodium citrate, 0.1% Triton X-100). The samples were kept at 4C in the dark for at least 30 min, dispersed by repeated pipetting before flow cytometry analysis in a FACS-Coulter Epics XL flow cytometer equipped with a single 488 nm argon laser. The percentage of nuclei in the different phases of the cell cycle (G_0_–G_1_, S and G_2_-M) was calculated with DNA cell cycle analysis software (Cell-Fit, Becton Dickinson). A minimum of 10^4^ cells/sample was analyzed for each sample.

### Western blotting

The intracellular concentration of TS protein was determined by Western blotting and immunodetection assuming 100% efficiency of both protein extraction and blotting (table 1). Western blot analysis was conducted as previously described [34]. Cells were harvested and washed twice in ice-cold 1×PBS, and resuspended in 20 mM Tris–HCl (pH 7.4), 150 mM NaCl, 1 mM EDTA (pH 8.0), 1% Triton X-100, and 0.1% SDS. Cells were lysed by freeze-thawing three times followed by sonication using three 2-to-3-s bursts. The insoluble debris was removed by centrifugation at 15,000×g for 30 min. 10 µg of each sample was resolved by SDS-PAGE (12%). The gels were electroblotted onto 100% pure nitrocellulose membranes (Amersham Hybond™-ECL™, GE Healthcare Bio-Science). Antibody staining was performed with an Infrared Dye detection system (LI-COR® IRDye®, LI-COR® Biosciences), using a 1∶500 dilution of the anti-human TS mouse TS106 monoclonal primary antibody (Abcam) in conjunction with a 1∶5000 dilution of IRDye® 800CW Conjugated Goat (polyclonal) Anti-Mouse IgG, highly cross adsorbed (LI-COR® Biosciences). Red Ponceau staining of the membrane prior immune-detection was used as loading control and to ensure equal efficiency of Western transfer (figure S3). Quantification of signal intensity was performed using LI-COR software (LI-COR® Biosciences). To determine the intracellular concentration of hTS, 10^5^ cells instead of 10 µg were resolved on SDS-PAGE and the absolute amount of TS protein was established using the standard curve obtained with purified TS protein (figure S1).

**Table 1:** Intracellular concentrations of TYMS mRNA and hTS.

	2008 cells			C13* cells		
	hTS Protein	TYMS mRNA	Ratio Prot/mRNA	hTS Protein	TYMS mRNA	Ratio Prot/mRNA
Intracellular concentration*	430±140 nM	3.6±0.9 nM	119	930±300 nM	10.3±2.6 nM	90
Pull down amount	139500±42000 amol	23±6 amol	6065	135000±37000 amol	45±5 amol	3000

* Using an estimated cell volume of 1.86*10^−12^ L.

Intracellular concentrations of hTS protein and TYMS mRNA are indicated. The amounts of hTS protein (figure S2) and TYMS mRNA in the pull down fraction after immunoprecipitation are also shown. Antibody against Beta-tubulin was used to evaluate the non-specific binding between TYMS mRNA and a generic protein. The non-specific interaction between mRNAs and hTS protein was checked by quantification of GAPDH mRNA bound to hTS in the pull down fraction. (Intracellular concentration: n = 5, error = ±S.D. Pull down amount: n = 2, error = ±S.E.)

### Reverse transcription and real-time PCR analyses

Total RNA was extracted from the cultured cells using TRI reagent (Sigma-Aldrich). Reverse transcription was performed with 2 µg of total RNA using random primers (Promega) and M-MLV reverse transcriptase (Promega). Real time RT–PCR was performed with 10 ng of cDNA using Power SYBR® Green PCR Master Mix (Eurogentec) and a Mini-Opticon™ (Bio-Rad), followed by dissociation curve analysis and subsequent agarose gel electrophoresis to confirm specificity of amplification. The following primer sets were used: TYMS [Genbank: NM_001071.1], forward: 5′-CAGATTATTCAGGACAGGGAGTT-3′, reverse: 5′-CATCAGAGGAAGATCTCTTGGATT-3′; GAPDH [Genbank: NM_002046.3], forward: 5′-CAAGGTCATCCATGACAACTTTG-3′, reverse: 5′-GGGCCATCCACAGTCTTCTG-3′; TYMS pre-mRNA [Genbank: NT_010859.14], forward: 5′-CCCTTCAGCTCTGATGGAAG-3′, reverse: 5′-GTTTCTGCAGGTGTCCATT-3′; p53 [Genbank: NM_001126112.1, NM_001126113.1, NM_001126114.1, NM_001126115.1, NM_001126116.1, NM_001126117.1, NM_000546.4] forward: 5′-CCCCAGGGAGCACTAAGCGAGCACT-3′,reverse: 5′-TCGAAGCGCTCACGCCCACGGA-3′. The amount of target, normalized to an endogenous reference (GADPH) and relative to a calibrator (2008 cell line or untreated sample), was given by 2^−ΔΔCt^ calculation [35]. To determine the intracellular concentration, the cDNA derived from 250 cells instead of 10 ng was quantified by Real Time PCR and the absolute amount of TYMS mRNA was established using a TYMS cDNA standard curve [36]. All experiments were carried out in triplicate; amplification plots were analyzed using the CFX Manager Software v 1.6 (Bio-Rad).

### Immunoprecipitation assay

Immunoprecipitation of TS-RNP complexes was performed as described by Peritz at al. [37] using protein A and TS monoclonal antibody (TS 106), which were recently used to investigate Zebrafish TS protein interaction with its own mRNA [38]. In brief, 5×10^6^ cells were harvested and washed twice in PBS. The cells were subsequently lysed in polysome lysis buffer (100 mM KCl, 5 mM MgCl_2_, 10 mM HEPES, pH 7.0, 0.5% Nonidet P-40, 1 mM DTT, 100 U ml^−1^ RNasin RNase inhibitor (Promega), 2 mM vanadyl ribonucleoside complex solution (Sigma-Aldrich), 25 µl ml^−1^ protease inhibitor cocktail for mammalian tissues (Sigma-Aldrich) and pre-cleared by two 1 h washes with protein A agarose beads 12.5% (GE healthcare Bio-Science) at 4°C. The cleared extract was then incubated with TS monoclonal antibody (TS 106) overnight at 4°C and the day after protein A agarose beads 12.5% were added for 6 h at 4°C. Immunoprecipitates were centrifuged at 250 *g* for 5 min and then washed four times with polysome lysis buffer. The pellets were subsequently solubilized in glycine 0.1 M pH 3 and used for both Western immunoblotting and RT-real time PCR analysis. The percentages of TYMS mRNA bound to TS protein in cell free extract and immunoprecipitates are reported where indicated. GAPDH mRNA and β-tubulin antibody (Abcam) were used to estimate the non-specific binding. The supernatant fraction after immunoprecipitation was used to exclude any effect due to the TYMS mRNA stability under the experimental conditions used.

### Drug interaction analyses

The effects of drug combinations were quantified by a synergism quotient (SQ) [39], [40]. The synergism quotient was defined as the net growth inhibitory effect of the drug combination divided by the sum of the net individual analogue effects on growth inhibition. A quotient of >1 indicates a synergistic effect, while a quotient of <1 indicates an antagonistic effect and a quotient close to 1 indicates an additive effect.

## Results

### Effect of 5-FU on TS expression

**Figure 1:**
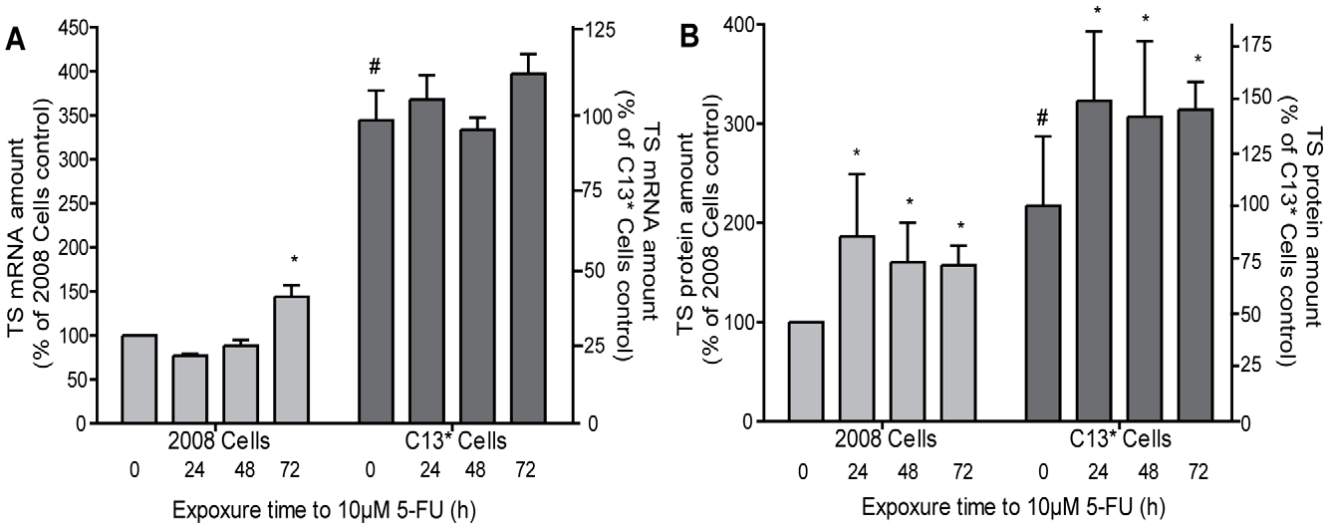
Modulation of TS mRNA and TS protein levels by 5-FU in 2008 and C13* cells. The amount of TS mRNA was determined by relative Real Time PCR using GAPDH mRNA as internal reference (panel A), while the amount of TS protein was determined by Western Immunoblot analysis at different times after addition of 5-FU. Red Ponceau staining of the membranes prior to immune-detection was used as loading control [66] (panel B). For each selected time the ratio between the TS level in presence and absence of 5-FU is shown. Results represent the mean of three separate experiments. Statistical significance was estimated by two-tailed unequal variance Student's t-test comparing either treated and untreated samples (*P<0.05, n = 3) or C13* cells and 2008 cells (# P<0.05, n = 5). Error bars indicate S.D.

We first investigated the effect of 5-FU on both the TS protein and the TS mRNA levels in the cisplatin/5-FU-resistant (C13*) and sensitive (2008) cell lines. We confirmed that the basal levels of both, TYMS mRNA and hTS protein are significantly higher in C13* cells compared to the 2008 parental cell line (p<0.05, n = 5, figure 1). Our time-course study indicated that treatment with 5-FU increased the TS mRNA level in the 2008 cells by 1.5-fold 72 hours after addition of 5-FU (p<0.05, n = 3, figure 1A). A modulation of the mRNA level was not observed in the sensitive cells after 24 h- and 48 h treatment (figure 1A); although the protein level was approximately two fold increased already 24 hours after treatment (p<0.05, n = 3, figure 1B). We also noticed that the sensitive 2008 cells showed a significant increase in protein levels after 5-FU treatment compared to the resistant cells, thus overcoming the low basal expression levels of TS protein. The 5-FU resistant C13* cell line also showed a level of TYMS mRNA 3.5-fold higher than the 2008 cell line (p<0.05, n = 5, figure 1A). Although, the level of TYMS mRNA in C13* cells does not appear to change during 5-FU treatment, we observed a 1.5-fold increase of the protein level in the presence of 5-FU (p<0.05, n = 3, figure 1B). Thus, during 5-FU treatment the steady-state expression level of TS protein increased in both cell lines, in part independently of transcriptional activity (figures 1A and 1B).

### TS mRNA regulation: synthesis, splicing and stability

**Figure 2:**
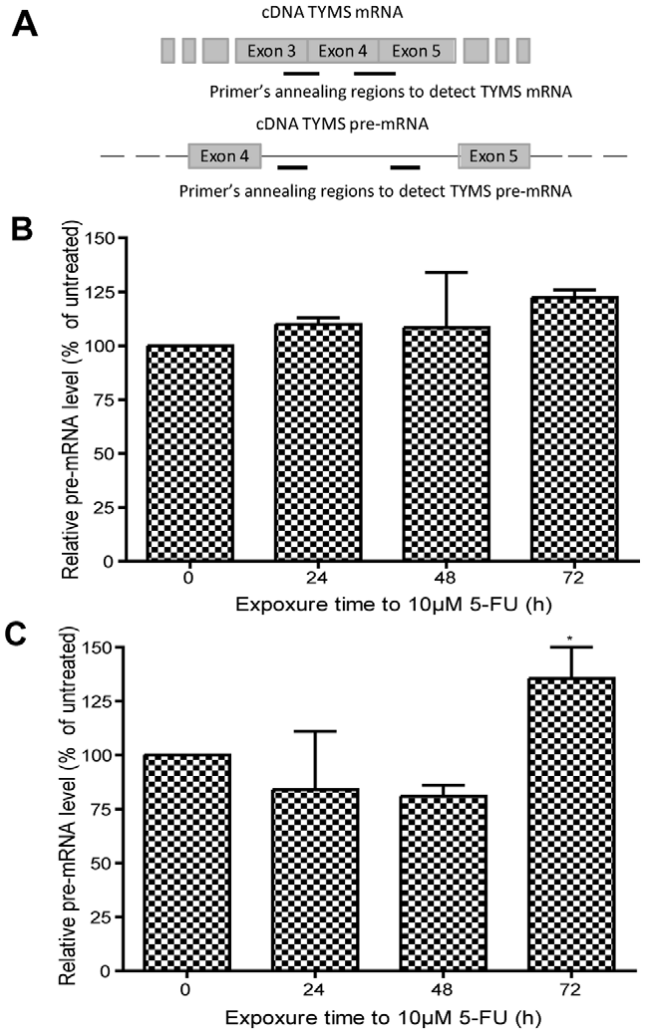
Measurements of TS pre-mRNA. Schematic representation of the annealing regions of the 2 primer couples used for TYMR mRNA and TYMS pre-mRNA measurements (panel A). The amounts of TS pre-mRNAs containing intron 4 were determined in 2008 cells (panel B) and C13* cells (panel C) by Real-Time PCR at different times after adding 5-FU. GAPDH mRNA was used as internal reference for Real Time PCR. For each selected time the ratio between the mRNA level in presence and absence of 5-FU is shown. Results represent the mean of three separate experiments. Statistical significance was estimated by two-tailed with unequal variance Student's t-test comparing treated samples with time 0 for each selected time (*P<0.05, n = 3). Error bars indicate S.D.

**Figure 3:**
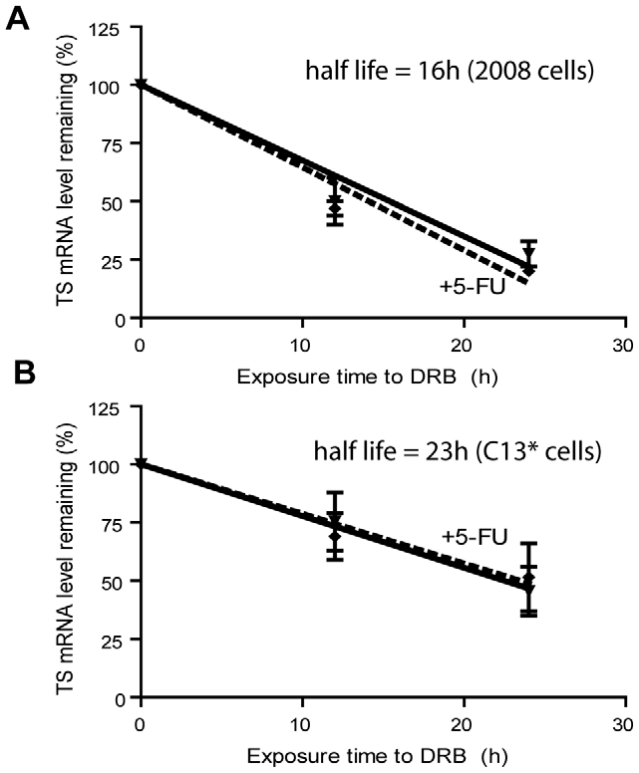
Measurements of TS mRNA stability. The level of TS mRNA was determined in 2008 cells (panel A) and C13* cells (panel B) by Real Time PCR at different times after the addition of DRB (inhibitor of RNA pol II). Results represent the mean of three separate experiments. Statistical significance was estimated by two-tailed unequal variance Student's t-test comparing samples treated with DRB with samples treated with DRB+5-FU at each selected time, (n = 3). Error bars indicate S.D.

As 5-FU is also directly incorporated into RNA, this pro-drug affects not only DNA synthesis, but also the RNA metabolism, pre-mRNA synthesis and processing, as well as mRNA stability [11]. In particular, it has been reported that incorporation of 5-FUTP in spliceosomal snRNA does block splicing [41]. To obtain more detailed insight into the action of 5-FU, we examined the TS pre-mRNA levels during 5-FU treatment by real time PCR using a primer couple that spanned the intron 4 of the human TYMS (figure 2A). Up to 48 h post treatment we did not find evidence for a significant modulation of the amount of TS pre-mRNA (n = 3, figures 2B and 2C). Subsequently the level of pre-mRNA carrying the intron 4 was 40% increased in C13* cell lines (p<0.05, n = 3, figure 2C), likely due to increased transcriptional activity of the TYMS gene (figure 1A). We also tested if the observed changes in hTS expression could result from differences in turnover of mRNA. Using 5,6-Dichlorobenzimidazole 1-β-D-ribofuranoside (DRB), a selective inhibitor of RNA polymerase II (RNA pol II) that blocks mRNAs synthesis completely, we found that the TS mRNA half-life was about 23 h and 16 h (p<0.05, n = 3, figure 3) in C13* and 2008 cells, respectively. This indicates increased stability of mRNA in the resistant cell line, which contributes to the different amounts of basal TS mRNA in the two cell lines (figure 1A). Treatment with 10 µM 5-FU did not affect the TS mRNA degradation rate, as the same half-life of TS mRNA was found in 5-FU treated and non-treated cultures (figures 3A and 3B).

### Cell cycle perturbation and p53 mRNA level

**Figure 4:**
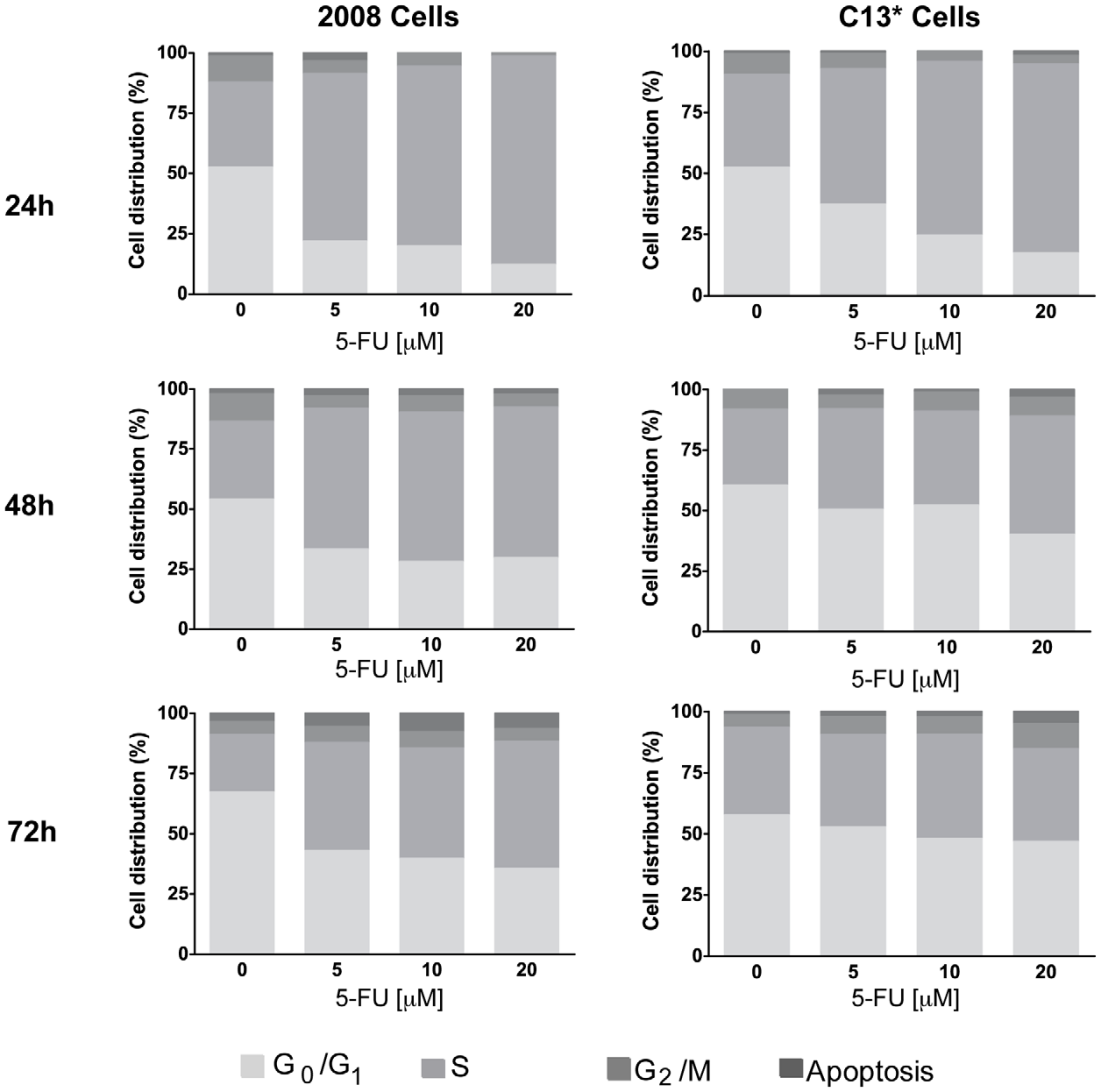
The effect of 5-FU on the cell cycle phase distribution of 2008 and C13* cells. Biparametric analysis based on flow cytometric analysis of the DNA content by PI staining and BrbU incorporation in 2008 and C13* cells is shown. After 24 h (top) 48 (middle) 72 h (down) of incubation with the indicated concentrations of 5-FU, cells were processed according to materials and methods. Similar results were obtained in three separate experiments. The error bars are omitted for a clearer visualization and standard deviations (SD) are reported in table S1 (n = 3).

It is well-known that 5-FU causes perturbation of the cell cycle phase distribution in many cell lines [14], [15], [42]. This is mainly due to the modulation of several protein families: cyclins, p-53 target genes and apoptosis regulatory pathways [43], [44]. Moreover, TYMS gene expression is modulated during the cell cycle since LSF, a transcription factor essential for stimulating G_1_-S gene expression, mediates the activation of the TYMS gene in the late G_1_ phase [24]. Consequently, we decided to investigate the cell cycle phase distribution during 5-FU treatment and to compare TYMS expression with the cell cycle perturbation. Our bi-parametric flow cytometry analyses using propidium iodide (PI) and an antibody against-BrdU, showed that 5-FU caused a significant perturbation of the cell cycle in both cell lines, with an accumulation of cells in the S phase and a substantial decrease of the cells in the G_0_–G_1_ and G_2_-M phases (n = 3, figure 4 and table S1). The sensitive 2008 cells showed a higher rate of perturbation of the cell cycle when compared with the resistant C13* cell line. In particular, after 24 h-treatment, the 2008 cells showed a dose-dependent increase of 2–3 fold of the cell number in the S phase, and a dose-dependent decrease from 2 to 5-fold of the cell number in both, G_1_–G_0_ and G_2_-M. At 48 h-treatment a partial recovery of the cell cycle distribution is observed, in particular at the highest concentration of 5-FU. Indeed, only a 2-fold reduction of the cell amount in both, G_0_–G_1_ and G_2_-M phases together with a 2-fold increase of cells in the S phase was observed in the presence of 20 µM 5-FU. At 72 h of treatment of 2008 cells with 5-FU, the cell amount was restored in the G_2_-M phase, whereas only a partial retrieval of the cell distribution between G_1_–G_0_ and S phase was observed. A pronounced 3–4 fold reduction of cells in the G_0_–G_1_ phase was also observed in the C13* cells after 24 h of treatment together with a 1.5–2 fold increase of the cells in the S phase and a 50% of reduction of the G_2_-M phase compared to the untreated cells. However, already after 48 h-treatment we observed roughly the same amount of cells in the G_2_/M phase in treated and untreated samples and, when compared with the 24 h-treatment, less perturbations of the cell distribution between S and G_1_–G_0_ phases. Finally, at 72 h of 5-FU treatment, the cell cycle profiles of the C13* cells were comparable with the control sample with regard to the cell distribution between S and G_1_–G_0_ phase and a visible G_2_-M block was observed at the higher concentration of 5-FU (20 µM). This observation agrees with the dual antitumor effect of 5-FU on the cell cycle [45] and indicates that at high concentrations 5-FU perturbs G_1_-S phases, whereas at lower concentrations this drug perturbs G_2_-M phases.

**Figure 5:**
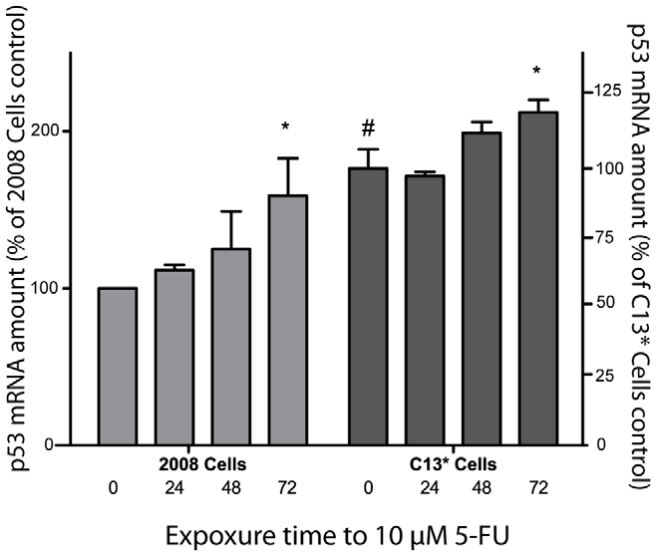
Modulation p53 mRNA by 5-FU in 2008 and C13* cells. The amount of p53 mRNA was determined Real Time PCR at different times after adding 5-FU. For each selected time the ratio between the p53 mRNA level in presence of 5-FU and p53 mRNA level in absence of 5-FU is shown. GAPDH mRNA was used as internal reference for Real Time PCR. Results represent the mean of three separate experiments. Statistical significance was estimated by two-tailed unequal variance Student's t-test comparing either treated samples with time 0 for each cell line (*P<0.05, n = 3) or C13* cells with 2008 cells (# P<0.05, n = 3). Error bars indicate S.D.

Since 5-FU provokes not only the inhibition of hTS, but also DNA and RNA damage and cDDP is a DNA-damaging agent (for a review see [11] and [46], respectively), we tested the possibility that p53 could be up-regulated in the cDDP-resistant 5-FU-cross-resistant cell line, C13*, with respect to the 2008 cells. The first analyses of p53 in these cell lines, performed using real time PCR, revealed that even in the absence of 5-FU treatment, the p53 transcript level was 2-fold higher in cells resistant to cisplatin/5-FU compared to sensitive ones (p<0.05, n = 3, figure 5). Up to 48 hours after treatment with 5-FU, the p53 transcript levels were constant in both cell lines. However, at the later time points an up-regulation of p53 mRNA of 1.5 and 1.2 fold was observed in both 2008 and C13* cells, respectively (p<0.05, n = 3, figure 5).

### Different schedules of drug treatment and anti-tumor responses

Since TYMS transcription occurs during both G_1_ and S phases [24], [47] and 5-FU increases the cell amount in the S phase, we decided to evaluate and compare the anti-tumor responses generated by different combinations of 5-FU and others agents which have shown the ability to compromise the cell cycle such as cDDP and the polyamine analog N1,N11-diethylnorspermine (DENSpm). It is well known that cDDP arrests the cell cycle in the G_2_/M phase [48] while DENSpm first retards the S phase progression and later increases the sub-G_1_ population and/or arrests the cell cycle in G_1_ [49], [50], [51], [52]. Thus, both drugs can impair the S phase and moreover have already shown a synergistic cell killing effect when combined with novel folate cycle inhibitors with quinoxaline structure in these ovarian carcinoma cell lines [20], [39]. Our results show that the cell killing resulting from the drug combinations between 5-FU and cDDP or DENSpm is affected by the drug treatment schedule (p<0.05, n = 3, table 2).

**Table 2:** Effects of sequential combination of 5-FU with cDDP and DENSpm on SQ values in 2008 and C13* cells.

2008 cells		Synergism Quotient	SD	C13* cells		Synergism Quotient	SD
Day 1	Day 2			Day 1	Day 2		
1 µM cDDP	2 µM 5FU	1.50^a^	0.14	4 µM cDDP	5 µM 5FU	0.57	0.21
2 µM 5-FU	1 µM cDDP	1.03^a^	0.19	5 µM 5FU	4 µM cDDP	0.65	0.19
3 µM cDDP	2 µM 5-FU	0.96	0.10	8 µM cDDP	5 µM 5FU	0.67	0.12
2 µM 5-FU	3 µM cDDP	0.96	0.20	5 µM 5FU	8 µM cDDP	0.71	0.10
2 µM DENSpm	2 µM 5-FU	1.84^b^	0.30	2 µM DENSpm	2 µM 5-FU	1.24^d^	0.08
2 µM 5-FU	2 µM DENSpm	1.23^b^	0.13	2 µM 5-FU	2 µM DENSpm	1.05^d^	0.08
4 µM DENSpm	2 µM 5-FU	1.35^c^	0.05	4 µM DENSpm	2 µM 5-FU	0.94	0.06
2 µM 5-FU	4 µM DENSpm	1.08^c^	0.06	2 µM 5-FU	4 µM DENSpm	0.94	0.10

Synergism of growth inhibition was determined by treatment of cells with 5-FU, DENSpm and cDDP alone and in sequential combination where the first drug was added at Day 1 and the second drug was added at Day 2. Counting the cell biomass was done at Day 4. Synergism Quotients (SQ) have been calculated as reported in material and methods. The concentration of 5-FU was chosen to obtain values for percentage growth inhibition no greater than 30% when added alone. Statistical significance was estimated by two-tailed paired Student's t-test comparing the samples where 5-FU was added before the other drug with the samples where 5-FU was added after the other drug. (^a,b,c,d^ P<0.05, n = 3, error = standard deviation (SD)).

In particular, in 2008 cells we observed a synergistic effect when either 1 µM cDDP or DENSpm are added 24 h before the treatment with 2 µM of 5-FU. When 5-FU was added either before cDDP or before DENSpm, we found no evidence for a strong synergistic effect. It is interesting to note that not only the timing, but also the concentration of the two agents can affect the outcome of the drug combination as underlined by the additive effect shown by the sequentially combination between 3 µM of cDDP and 2 µM of 5-FU in the sensitive cells (table 2). In the C13* cell line, in agreement with the cDDP-resistance and 5-FU-cross-resistance, all the combinations between 5-FU and cDDP result in an antagonistic effect. On the other hand, also here a synergistic effect is observed when 2 µM of the polyamine analog is added 24 h before 5-FU. The other drug combinations between 5-FU and DENSpm are additive, confirming that the cell killing can be affected by different schedules of drug combination.

### TS protein regulation: translational repression

We have shown earlier that 5-FU increases the stability of hTS in the cell lines used in this study [20]. To investigate in our cell lines also the validity of the auto-regulatory model, postulating that translation of TYMS mRNA is controlled by hTS itself (reviewed in [19]), we evaluated the amount of TS mRNA bound to hTS protein in whole cell extracts. Using quantitative RT-PCR and Western immunoblot assays, we estimated the intracellular concentrations of mature TYMS mRNA and hTS protein (n = 5, table 1). In agreement with earlier work performed in HuTu 80, HT-29 and WIDR cell lines [21], [53], we found that the intracellular concentration of hTS protein is 0.43±0.14 µM in 2008 cells and 0.93±0.3 µM in C13* cells (p<0.05, n = 5, table 1), while the mature TYMS mRNA is 3.6±0.9 nM and 10.3±2.6 nM (p<0.05, n = 5, table 1), respectively. This indicates that the hTS protein is present in considerable (approximately 100-fold) molar excess in the cytosol. This ratio may nevertheless be an overestimation, as hTS is expressed up to 60% [18] in the nucleus [1], [54] and also in mitochondria [2]. We also investigated the amount of mRNA in hTS immuno-precipitates (table 1 and figure S2). The ratio between hTS protein and TYMS mRNA observed in the pull-down fractions after immunoprecipitation was significantly higher than in the cell, suggesting that TYMS mRNA did not significantly co-precipitate with hTS under these experimental conditions. After correcting for non-specific RNA binding with an unrelated antibody, we estimated that only 2.0%±1.9 and 3.0%±1.6 of TYMS mRNA were bound to TS protein in 2008 and in C13* cells.

### TYMS transcription is required for up-regulation of hTS by 5-FU

**Figure 6:**
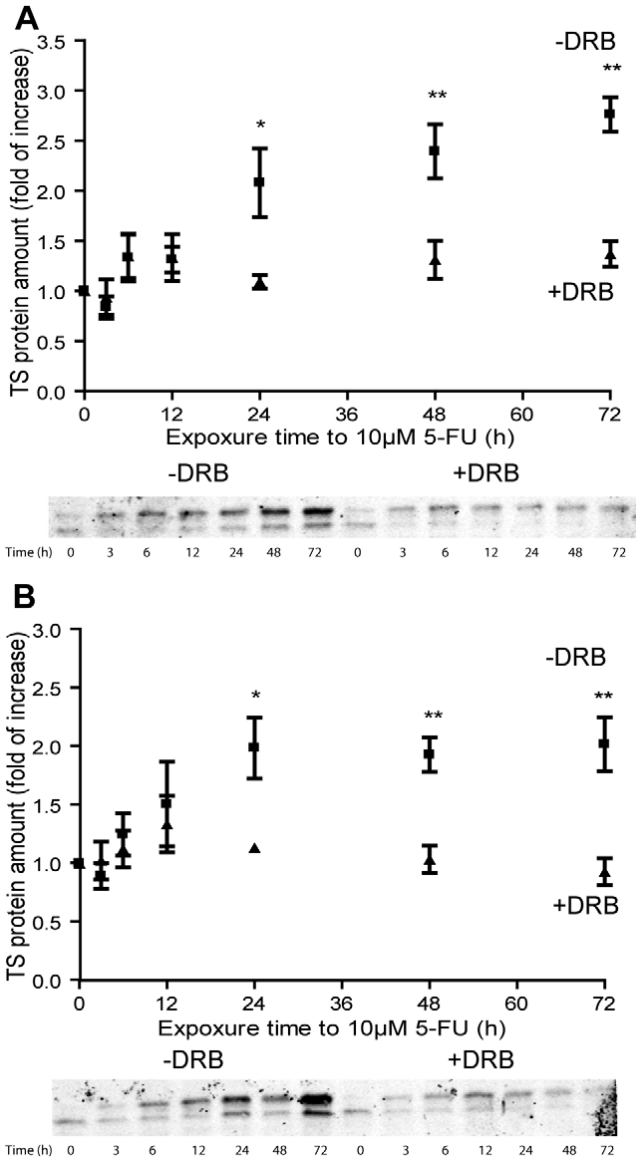
Role of TS mRNA synthesis in TS protein up-regulation. The concentration of TS protein in 2008 (panel A) and C13* cells (panel B) was determined by Western blotting at various times after the addition of 5-FU to the control media (closed squares) and DRB (inhibitor of RNA pol II)-treated cells (closed triangles). For each time selected 10 µg of protein extract was resolved in SDS-PAGE (12%) and the TS protein amount is given with respect to time 0. Results represent the mean of five separate experiments. Red Ponceau staining of the membranes prior to immune-detection was used as loading control [66] (Figure S3). Immunodetection of typical experiment is shown in the bottom part of each panel. Statistical significance was estimated by two-tailed with unequal variance Student's t-test comparing samples treated with 5-FU with samples treated with 5-FU+DRB at each selected time (*P<0.05; **P<0.01, n = 5). Error bars indicate S.D.

To investigate to which extent transcriptional activity is required for up-regulation of hTS in 5-FU treated cells, we also determined TS protein levels in DRB-treated and untreated control cells. The inhibition of RNA pol II by DRB was confirmed using qPCR analysis of TS mRNA levels under the same conditions used for protein determination. The data reported in figures 6A and 6B show that up to 12 hours after 5-FU treatment, no obvious difference in TS protein levels was detected in samples pre-treated with DRB or in negative controls in 2008 or C13* cell lines (n = 5). On the contrary, after 24-h treatment, the amount of TS protein was significantly lower in cells pre-treated with DRB when compared with non-treated control cells (p<0.05, n = 5, figure 6). As the turnover rate of the ribosome is reported to be between 4 and 10 days [55], [56], [57], we can exclude any significant effect due to newly synthetized ribosomes in our data set. Thus, our data support the hypothesis that during the first 12 hours after treatment with 5-FU, the increase in TS protein is due to the translation of TS mRNA synthesized before addition of 5-FU and/or increased stability of the ternary complex. After this initial period, it is transcription that is responsible for the increased expression level of hTS in 5-FU treated samples. Our data also suggest that the inhibition of hTS activity and up-regulation of hTS protein levels are not directly linked, as the inactivated ternary complex was detected already 3 hours after 5-FU exposure, whereas a substantial increase in global TS levels was detected only after 24 hours (figures 6A and 6B).

## Discussion

In this study we investigated the regulatory mechanisms that bring about up-regulation of hTS in 5-FU treated cells using the ovarian cancer cell line 2008 and the corresponding cisplatin-resistant and 5-FU cross-resistant subline C13*. Our results indicate that quantity, splicing and stability of TYMS mRNA are not significantly altered during the first 48 h of 5-FU treatment (figures 1, 2 and 3). In addition, we have demonstrated that the basal transcription of TYMS mRNA is the key factor required for up-regulation of hTS in drug-treated ovarian cancer cells. For instance, we have shown that the RNA polymerase II inhibitor DRB substantially limits up-regulation of TS proteins after addition of 5-FU, indicating that 70–80% of the up-regulation depend on the *de novo synthesis* of TYMS mRNA in 2008 and C13* cells, respectively (figure 6). The TYMS mRNA already present before addition of 5-FU contributes to up-regulation of the hTS protein during the first 12 hours of treatment (figure 6), albeit to a lower extent than observed at the later time points (see above).

Moreover, the presence of the inactive ternary complex 3–6 hours posteriori of 5-FU addition (figure 6) supports the notion that the steady-state expression level of hTS reflects increased stability of hTS in the ternary complex, in agreement with earlier observations indicating that hTS enzyme stability is linked to a conformational change due to the presence of the substrates [58]. In contrast, the amount of the TYMS mRNA – hTS complex observed in these ovarian cancer cell lines (table 1) is very small and not fully consistent with a translational de-repression mechanism proposed earlier for the up-regulation of human TS by 5-FU in other cell lines [26], [27], [28]. Our quantitative data suggest that in whole cell extracts at least 95% of TYMS mRNA present in the cell were not bound to hTS protein with high affinity, even under the reducing conditions (1 mM DTT) that have been reported to enhance the RNA binding activity of hTS [59]. The formation of the hTS-RNA complex should have been favored by a molar excess (up to 100-fold) of hTS over its own cytosolic mRNA (table 1). Thus, our results indicate that enzyme stabilization [20], rather than translational de-repression, is one of the pivotal mechanisms in hTS protein accumulation. Accordingly, the increase in protein levels in 2008 cells during 5-FU treatment correlates with the major increase in hTS protein stability found in this cell line when compared with the resistant one. In particular, in the presence of 5-FU the hTS half-life is increased 2.5 fold (from 6 h to 15 h) in 2008 cells and only 1.9 fold (from 11 h to 21 h) in the resistant C13* cells [20].

Notably, cell cycle analyses reveal that 5-FU compromises the cell cycle by blocking the 2008 cell line in the S phase (figure 4). It is known that TYSM mRNA is synthesized in the S phase of the cell cycle, as well as in the G_1_ phase [24], [47]. After the pronounced S phase block in 2008 cells at 24 h treatment, we observed a slight recovery of the cell cycle distribution after 48 h treatment and a more evident restoration at 72 h, together with an increase in TYMS transcript. Similar results have been obtained also for the C13* cells, although, in agreement with the resistant phenotype, this cell line showed a faster restoration of the cell cycle distribution and overcomes the S phase block at 72 h of treatment. This effect is only partially observed in 2008 cells, which are 2.5 fold more sensitive to 5-FU. This is demonstrated by IC_50_ values for 5-FU of 3.5 µM and 8.2 µM that were determined by a cell growth inhibition assay at 72 h post-treatment in 2008 cells and C13* cells, respectively [20]. This higher resistance to 5-FU could result from increased detoxification of the drug [60], [61] and more active DNA synthesis and repair processes [62], [63] together with increased expression of the folate cycle enzymes, thymidylate synthase and dihydrofolate reductase, due to the cisplatin-resistance phenotype [20]. We have also shown that p53 is transcribed constitutively at two-fold higher level in the resistant cells when compared to a parental cell line (figure 5), in agreement with an up-regulation of DNA synthesis and repair mechanisms. It is also of interest that the increase in TYMS transcription (figure 1A and 2C) occurs after the changes in the cell cycle distribution, which have been observed already after 24 h-treatment (figure 4), further indicating that increase in TYMS transcription is a relatively late cellular response to 5-FU. This enhanced transcription of TYMS could be part of a more complex pathway involving p53, as transcriptional up-regulation of p53 and TYMS are at least partially temporally linked (figures 1A, 2C and 5).

The fact that 5-FU has multiple cellular targets makes it difficult to fully establish causal links between the multiple and complex regulatory mechanisms affecting TYMS up-regulation. Nevertheless, our data has clearly established that the inhibition of hTS itself, 3 h-6 h after 5-FU addition, is not directly involved in the transcriptional activation, that occurs 72 hours after treatment, and only a later stimulus, characterized also by the increase of p53 mRNA levels, seems to be the starting point of the transcriptional activation. Since p53 is mainly involved in the pathways of DNA damage/repair, we hypothesize that the DNA damage could be one of the causes of this increase of the transcription of the TYMS gene. Moreover, it is of particular interest that recent reports have shown that the sub-cellular localization of hTS is cell cycle-dependent. In particular, in the S and G_2_-M phases, hTS is present in the nucleus to enable nuclear *de novo* synthesis of thymidylate during DNA replication and repair [1] and to prevent uracil accumulation in nuclear DNA [64] avoiding DNA damage. Our results suggest a direct link between TYMS transcription and cell cycle perturbation resulting from 5-FU treatment, as the TYMS gene is transcribed in the S phase where hTS is in the nucleus to overcome the cytotoxic effects provoked by metabolites of 5-FU. Thus, the S phase-block could promote cell survival in the presence of 5-FU through an increase in the level of hTS in the nucleus due to both, enhanced transcription and intracellular localization induced by the cell cycle.

Our findings also suggest that a combination of 5-FU together with cell cycle modulators may result in beneficial synergic drug effects. We showed that distinct anti-tumor responses are generated by different schedules of drug combination (p<0.05, n = 3, table 2). Pre-treatment with both cDDP and DENSpm is known to reduce or impair the S phase [48], [49], [50], [51], [52] and may explain the observed synergistic effects of these drugs with 5-FU in our cell models. Since we have observed a synergistic effect primarily when the cell cycle modulator (i.e. cDDP and DENSpm) is added 24 h before 5-FU, we suggest that a reduced or impaired S phase could increase the cytotoxic effect of the following administration of 5-FU. However, the cell cycle modulation of DENSpm is strongly time-dependent [52], possibly explaining why an antagonist effect between DENSpm and 5-FU was found when 5-FU was added 48 h after DENSpm in HCT116 colon cancer cells [51]. In any event, our combined findings suggest that the effects of the drug combinations between 5-FU and both DENSpm and cDDP are closely related to the drug treatment schedule.

Moreover, combinations of 5-FU and other drugs that have been reported in the literature, such as RPR-115135, a farnesyltransferase inhibitor, show a synergic effect on growth inhibition only in human colon cancer cell lines. Here the drug combination drastically reduces the amount of cells either in the G_0_–G_1_ or the S phase (HCT-116, LoVo, RKO, DLD-1, Colo-320), whereas an antagonism or a non-significant effect is observed when both G_0_–G_1_ and S phases are slightly modulated or increased (SW-620, HT-29, HCT- 15, KM-12) [42]. Moreover, the co-treatments with 5-FU and two inhibitors of histone deacetylase (HDAC), either Vorinostat or LBH589, decrease either the amount of cells in the G_1_ or S phase together with a inhibition of TYMS gene expression in colon cancer cells (HCT-116 and HT-29), therefore enhancing the effect of 5-FU [14]. Besides these synergic drug combinations acting on cell cycle distribution, it has been reported that Trichostatin A, another HDAC inhibitor synergic with 5-FU, affects the protein stability of hTS [65], confirming the important role of this mechanism in TS protein regulation. We also note that the cDDP/5-FU-resistant cells, which have high basal levels of both, TYMS mRNA and hTS protein, showed a behavior similar to the cDDP/5-FU-sensitive cell line. This suggests that key mechanisms regulating hTS expression are maintained even under conditions of high steady-state expression levels of hTS.

**Figure 7:**
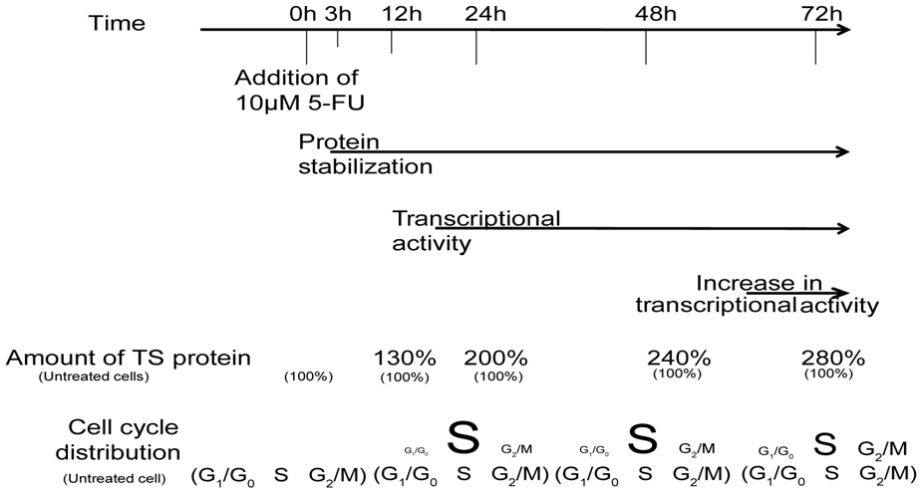
Schematic representation of hTS regulation in the 2008 cell line during treatment with 10 µM 5-FU. All the mechanisms involved in hTS regulation that have been observed in this work are shown with respect to the time scale indicated in the upper part. Continued arrows represent the time window where the phenomenon reported in the figure has been observed. TS protein amount is reported with respect to the untreated cells at the selected time. The percentages of cells in the different phases of the cell cycle are proportional to the size of the letters indicating the different phases.

In summary, our experimental data indicate that, constitutive TYMS mRNA transcription and cell cycle-induced hTS regulation (i.e. increasing the amount of cells in the S phase) together with hTS enzyme stability [20], are the three key mechanisms that mediate 5-fluorouracil induced up-regulation of human thymidylate synthase expression in the two ovarian cancer cell lines studied. We have also established that these three regulatory phenomena have a precise order (figure 7), suggesting the possibility of new therapeutic strategies based upon our findings.

## Supporting Information

Figure S1 **hTS protein standard curve for absolute quantification.** hTS protein standard curve has been obtained by serial dilution of purified hTS followed by SDS-PAGE, electroblotting, antibody staining (panel B) and quantization of signal intensity using LI-COR (panel A) as described in the material and method. The correlation coefficient is also shown (panel A).
(DOCX)

Figure S2 **hTS detection in the immunoprecipitation analysis.** The amounts of hTS protein after the immunoprecipitation (IP) assay were quantified by Western blot as described under materials and methods. Lane 1: supernatant of IP using hTS antibody in 2008 cells. Lane 2: pull down fraction of IP using hTS antibody in 2008 cells. Lane 3: pull down fraction of IP using Beta-tubulin antibody. Lane 4: supernatant of IP using hTS antibody in C13* cells. Lane 5: pull down fraction of IP using hTS antibody in C13* cells. Lane 6: pull down fraction of IP using Beta-tubulin antibody in C13 cells.
(DOCX)

Figure S3 **Red Ponceau staining of the western blot reported in** **figure 6** **.** Red ponceau staining prior immunodetection in 2008 cells (panel A) and C13* cells (panel B) was used as loading control and to confirm equal efficiency during Western transfer.
(DOCX)

Table S1 Standard deviations (SD) of the cell-cycle phase distribution (%). Standard deviations of the cell cycle distributions are shown for every phase of each sample reported in figure 4 (n = 3).
(DOCX)

### 
