# Supplementary figures and images for: Transcriptional Activation and Cell Cycle Block Are the Keys for 5-Fluorouracil Induced Up-Regulation of Human Thymidylate Synthase Expression
Source: PLoS One. 2012 Oct 9;7(10):e47318. doi: 10.1371/journal.pone.0047318 (PMC3467224; doi:10.1371/journal.pone.0047318)

**
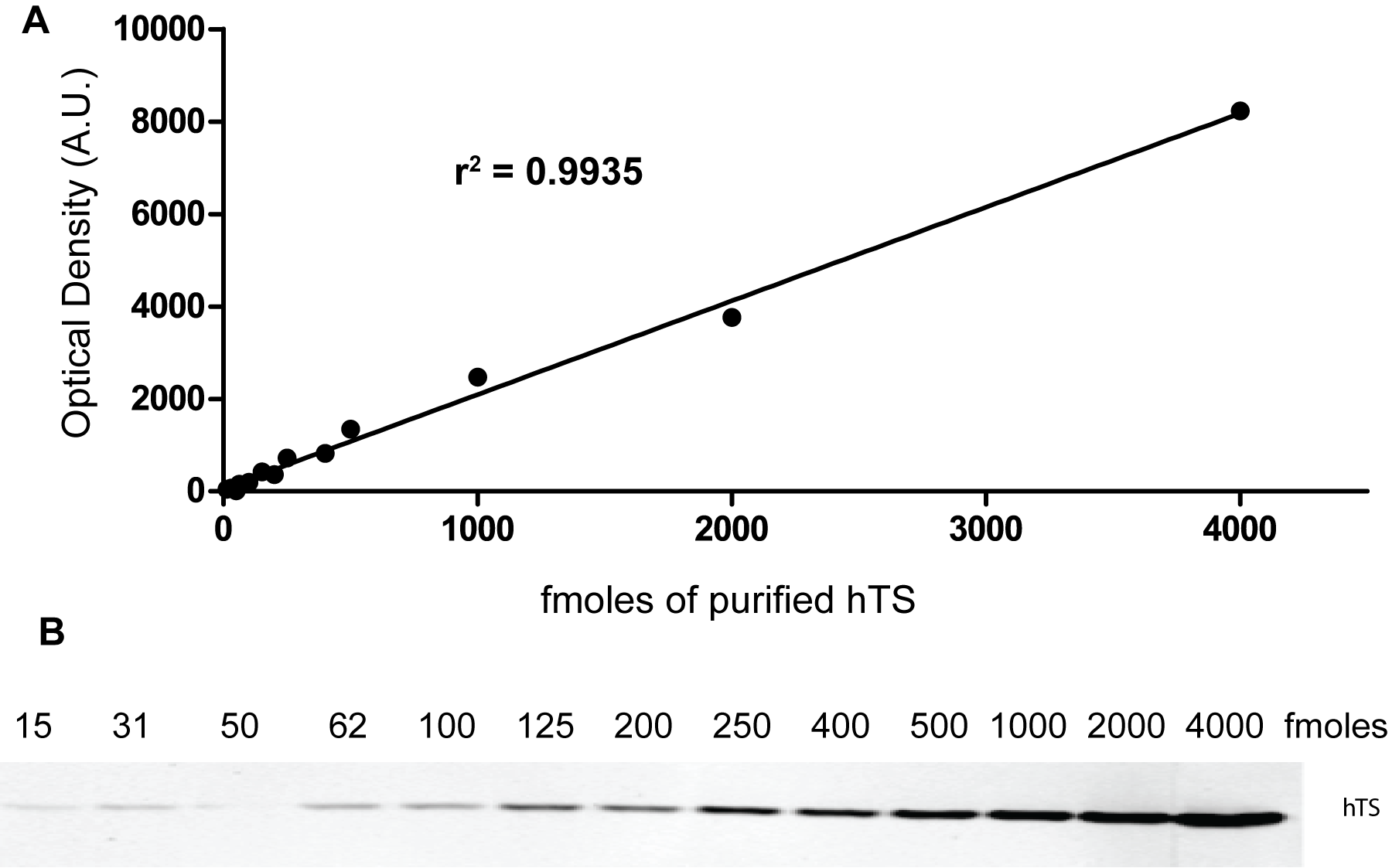
**

Supplement: Figure S1 — hTS protein standard curve for absolute quantification. hTS protein standard curve has been obtained by serial dilution of purified hTS followed by SDS-PAGE, electroblotting, antibody staining (panel B) and quantization of signal intensity using LI-COR (panel A) as described in the material and method. The correlation coefficient is also shown (panel A). (DOCX) [file pone.0047318.s001.docx]

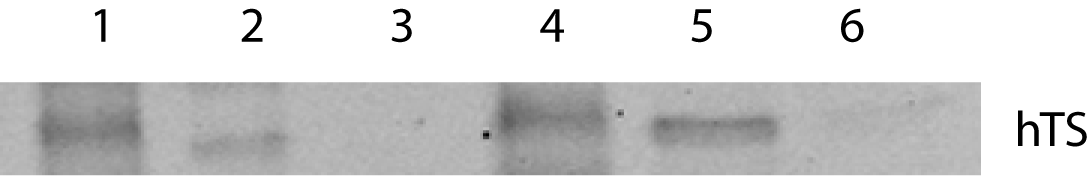

Supplement: Figure S2 — hTS detection in the immunoprecipitation analysis. The amounts of hTS protein after the immunoprecipitation (IP) assay were quantified by Western blot as described under materials and methods. Lane 1: supernatant of IP using hTS antibody in 2008 cells. Lane 2: pull down fraction of IP using hTS antibody in 2008 cells. Lane 3: pull down fraction of IP using Beta-tubulin antibody. Lane 4: supernatant of IP using hTS antibody in C13* cells. Lane 5: pull down fraction of IP using hTS antibody in C13* cells. Lane 6: pull down fraction of IP using Beta-tubulin antibody in C13 cells. (DOCX) [file pone.0047318.s002.docx]

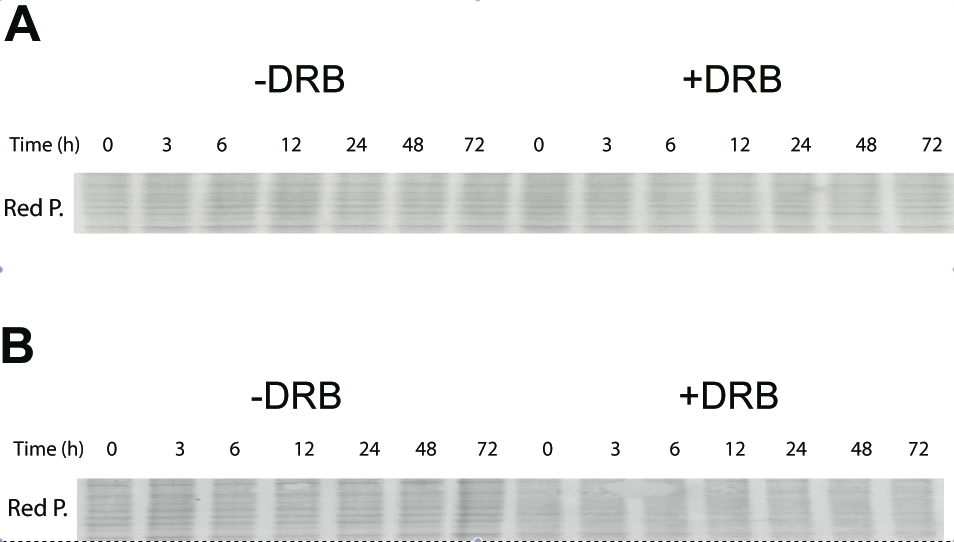

Supplement: Figure S3 — Red Ponceau staining of the western blot reported in figure 6 . Red ponceau staining prior immunodetection in 2008 cells (panel A) and C13* cells (panel B) was used as loading control and to confirm equal efficiency during Western transfer. (DOCX) [file pone.0047318.s003.docx]
