# Supplementary material for: Transcriptional Activation and Cell Cycle Block Are the Keys for 5-Fluorouracil Induced Up-Regulation of Human Thymidylate Synthase Expression
Source: PLoS One. 2012 Oct 9;7(10):e47318. doi: 10.1371/journal.pone.0047318 (PMC3467224; doi:10.1371/journal.pone.0047318)
Supplement: Table S1 — Standard deviations (SD) of the cell-cycle phase distribution (%). Standard deviations of the cell cycle distributions are shown for every phase of each sample reported in figure 4 (n = 3). (DOCX) [file pone.0047318.s004.docx]

**Table S1. Standard deviations (SD) of the cell-cycle phase distribution (%)**

|  |  | 2008 cells | | | | C13* cells | | | |
| --- | --- | --- | --- | --- | --- | --- | --- | --- | --- |
| Time | 5-FU µM | G_0_/G_1_ | S | G_2_/M | Apo | G_0_/G_1_ | S | G_2_/M | Apo |
| 24 h | 0 | 0.80 | 2.45 | 2.20 | 0.55 | 2.70 | 4.05 | 0.65 | 0.70 |
|  | 5 | 3.40 | 5.85 | 0.66 | 1.79 | 3.05 | 4.10 | 0.18 | 1.18 |
|  | 10 | 7.75 | 5.52 | 0.53 | 4.38 | 4.20 | 4.25 | 0.35 | 0.30 |
|  | 20 | 1.55 | 0.78 | 0.13 | 1,13 | 3,45 | 5,20 | 0,32 | 1,53 |
| 48 h | 0 | 2.62 | 2.76 | 2.55 | 1.25 | 8.85 | 9.45 | 1.25 | 0.65 |
|  | 5 | 2.62 | 3.75 | 1.86 | 1.06 | 8.25 | 10.65 | 1.02 | 1.39 |
|  | 10 | 3.04 | 3.89 | 0.53 | 1.13 | 6.52 | 8.35 | 1.35 | 0.48 |
|  | 20 | 13.55 | 15.06 | 0.57 | 0.94 | 3.30 | 4.80 | 0.76 | 0.75 |
| 72 h | 0 | 1.15 | 2.50 | 0.82 | 0.53 | 1.20 | 1.06 | 0.57 | 0.47 |
|  | 5 | 8.65 | 8.65 | 0.11 | 0.11 | 3.65 | 4.70 | 0.56 | 0.50 |
|  | 10 | 6.25 | 11.00 | 0.58 | 4.17 | 1.90 | 3.90 | 1.51 | 0.50 |
|  | 20 | 1.00 | 4.00 | 1.18 | 4.18 | 0.78 | 4.20 | 2.20 | 1.45 |

SD of the cell cycle distributions are shown for every phase of each sample reported in figure 4 (n=3).
